# Socio-cognitive determinants affecting insulin adherence/non-adherence in late adolescents and young adults with type 1 diabetes: a systematic review protocol

**DOI:** 10.1007/s40200-022-01054-8

**Published:** 2022-05-26

**Authors:** Hanan AlBurno, Liesbeth Mercken, Hein de Vries, Dabia Al Mohannadi, Stefan Jongen, Francine Schneider

**Affiliations:** 1 Care and Public Health Research Institute (CAPHRI), Maastricht University, Maastricht, The Netherlands; 2 Department of Health Psychology, Faculty of Psychology, Open University of The Netherlands, Heerlen, The Netherlands; 3 Faculty of Science and Engineering, Maastricht University, Maastricht, The Netherlands; 4 Department of Endocrinology and Diabetes, Hamad General Hospital, Doha, Qatar

**Keywords:** Socio-cognitive, Insulin, Adherence, Non-adherence, Type 1 diabetes, Adolescents, Young adults, Systematic review

## Abstract

**Objective:**

This systematic review aims to investigate the key socio-cognitive determinants associated with adherence/non-adherence to insulin treatment in late adolescents and young adults in the age range of 17–24 years with T1D.

**Methods:**

A pre-specified search strategy will be used to search for studies in the electronic databases and citation indexes: PubMed, EMBASE, Web of Science, and PsycINFO. Two researchers will screen the title and the abstract independently, then will read and critically appraise the full text of each included study. A third independent reviewer will resolve disagreements in data extraction until consensus. Data will be extracted using the Population, Exposure, Outcomes, Study characteristics framework. Study selection will follow the updated guideline for reporting systematic reviews (PRISMA 2020) and will take place from 15 October 2021 to 1 January 2022. The methodological quality and risk of bias of the observational studies will be assessed by the JBI Critical Appraisal Checklist for Cohort and JBI Critical Appraisal Checklist for Analytical Cross Sectional Studies.

**Results:**

A qualitative narrative synthesis will present the characteristics and the quality of studies and the outcomes of concern.

**Conclusion:**

Based on the contemporary literature, this review will synthesize the evidence on the socio-cognitive determinants associated with adherence/non-adherence to insulin treatment in late adolescents and young adults in the age range of 17–24 years with T1D. The findings will help design patient-centered interventions to promote adherence to insulin in this age group, guide patients’ consultations and diabetes self-management education (DSME) programs.

Protocol registration: PROSPERO ID: CRD42021233074.

## Introduction

Type 1 diabetes (T1D) is a global health problem with increasing prevalence at 3–5% yearly [1–3]. Although T1D sometimes appears during adulthood, it usually occurs during childhood or adolescence and is treated with insulin therapy. Despite the advancements in insulin administration systems, insulin adherence continues to pose a significant challenge for adolescents and young adults (AYAs) with T1D [4–7]. Adherence to insulin can be defined as administering the correct dose at the right time and/or frequency in accordance with a mutually agreed-upon treatment regimen [8, 9].

Evidence suggests that the rate of insulin non-adherence in AYAs is high [4, 10–12], ranging from 23—77%, with a higher rate in developing countries [13]. Research has demonstrated that non-adherence to insulin is associated with increased glycated hemoglobin (HbA1c) level [5, 14], diabetic ketoacidosis [15], increased hospitalization [16], and long-term complications [15–17]. The latter includes microvascular and macrovascular complications leading to increased morbidity and mortality in people  with T1D [4, 8, 17]. On the other side, the association between greater adherence and improved clinical outcomes in AYAs with T1D is evident [5, 6, 14]. Insulin non-adherence can occur intentionally or unintentionally and involves situations where adolescents and young adults did not fill their insulin prescriptions [17, 18], reduced or omitted doses [4, 7, 19]. The latter may occur for various reasons, such as fear of hypoglycemia [20], weight control [4, 7, 19], interference with daily routine, forgetfulness [13], problems with coping with peers [21], and social stigma [22, 23]. Sometimes AYAs unintentionally administered the wrong dose [21, 24]. Due to the negative (health) consequences of sub-optimal adherence, it is imperative to understand adherence/non-adherence behaviors and their factors.

Medication adherence is affected by multiple interacting factors [4, 25, 26]. Some of these factors are relatively fixed factors, such as socio-demographic (SD) factors (e.g., age, gender, ethnicity, personality, etc.) [14, 27–29] and socioeconomic (SE) factors (e.g., cost of treatment) [5, 27–29]. For example, findings revealed that AYAs were the least adherent and had poorer diabetes control compared with children and older patients [4–7, 18]. A systematic review among adolescents with T1D found that female sex was associated with non-adherence in more than one study [30]. Multiple studies involving T1D demonstrated eating disorders were more common in females than in males [31, 32]. The prevalence of eating disorders increased with age, affecting up to 40% of young adult females with T1D [33]. Eating disorders were found to be associated with lower insulin adherence and higher HbA1c [11, 19, 34]. Other factors are either difficult to modify, such as certain affect psychosocial factors (e.g., diabetes emotional distress, depression, anxiety, etc.) [14, 19, 30, 35] or partly unmodifiable such as medication regimen factors including the complexity of insulin regimen [5, 13, 36], type of administration devices [4, 14, 27], and insulin side effects (e.g., hypoglycemia).

**Fig. 1:**
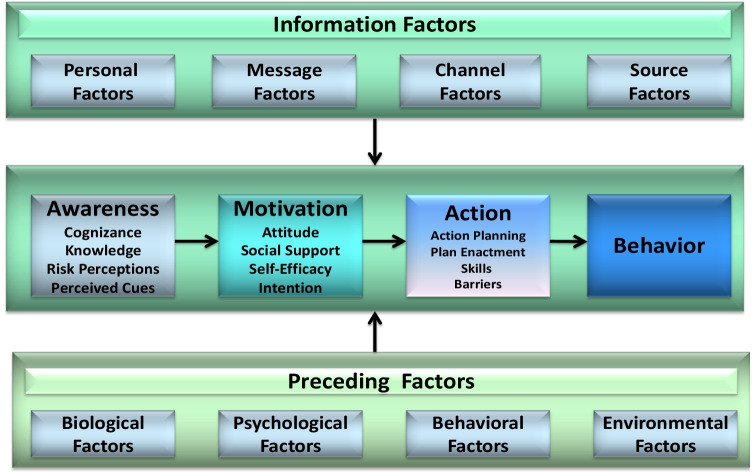
The I-Change Model. This figure has been reproduced with permission from de Vries H. An integrated approach for understanding health behavior; the I-change model as an example. Psychol Behav Sci Int J. 2017;2(2):555–85. https://doi.org/10.19080/PBSIJ.2017.02.555585

Certain factors, such as socio-cognitive factors, are, however, more likely to be modifiable [4, 26, 27]. Having insight into these modifiable factors can help to inform future interventions aimed at improving adherence through minimizing barriers and maintaining/promoting facilitators. There is a growing body of knowledge on the various psychological/ behavioral models used to examine the socio-cognitive factors that influence adherence to insulin [37]; still, all potentially relevant psychosocial factors were not yet considered in an integrated way [38]. Holmes and colleagues (2014) argue that within the theoretical models, researchers often focus explicitly on evaluating variables that are considered proximal (close) rather than distal to adherence behavior [39]. Hence, the I-Change model (ICM) [40] will be the leading theoretical frame for the present review (Fig. 1). The ICM integrates broader determinants of personal and environmental factors for the diagnosis of behavior, ranging from the individual ‘s degree of health literacy and knowledge to the social environment and setting for carrying out and maintaining the behavior. It distinguishes between pre-motivational factors (cognizance of one's  behavior, knowledge, risk perceptions, and cues to action), motivational factors (attitude, social support, self-efficacy, and intention), post-motivational factors (action and coping planning), and distal information factors. This is particularly relevant for diabetes control as multiple empirical studies have shown that sets of interactively integrated factors account for variations in adherence to the prescribed recommendations [25, 41, 42].

Several studies have investigated and identified important socio-cognitive factors within adolescents and/or young adults. Some were related to pre-motivational factors such as knowledge and expectancies [43, 44], perceived severity of the disease, perceived susceptibility or vulnerability to the disease process, perceived barriers/costs to the action, and cues to action [45, 46]. Regarding motivational factors, results by de Weerdt and colleagues (1990) showed that attitude was an essential determinant of active self-care of AYAs along with features of their social environment [47]. Other studies showed positive correlations of perceived self-efficacy and outcome expectancies with insulin adherence [48, 49]. Others identified the role of the social influence of family, peers,  and  the healthcare system on adherence [50–52]. Within the post-motivational factors, past studies demonstrated that the coping mechanism and appraisal of coping/ progress would modify the representation and/or coping behaviors [53, 54]. Regarding distal information factors, studies highlighted considering features such as level, frequency, type, relevance to the recipient, and quality of information to provide personalized information to AYAs  [55, 56], and how the lack of information has impacted diabetes management negatively [57].

Systematic reviews that looked at socio-cognitive determinants of insulin adherence among people with T1D mainly focused on adults (i.e., Sigurdardóttir et al., 2005; Gherman et al., 2011; Davies et al., 2013) [27, 58, 59]. The systematic/narrative reviews that did include adolescents and /or young adults did not exclusively relate to insulin adherence (investigated adherence to a range of diabetes self-management behaviors including diet, physical activity, self-monitoring of blood glucose and medication adherence (i.e., Coyle et al., 2013; Neylon et al. 2015; Martinez et al., 2016) [28, 30, 60] and/or did not exclusively relate to T1D (included patients with either type 1 or type 2 diabetes), (i.e., Nagasawa et al. 1990; Coyle et al., 2013; Gonzalez et al., 2016; Robinson et al., 2021) [29, 60–62] and/or focused mainly on a few determinants (i.e., Young et al., 2013; Datye et al., 2015; Neylon et al. 2015) [5, 28, 63]. Hence, the relevance of these findings for AYAs with T1D is unclear. One narrative review [5] specifically addressed insulin adherence in adolescents with T1D, considered mainly psychological factors (e.g., mood, anxiety, and eating disorders), social support factors, and interactions with healthcare system factors.

To date, a wide range of other socio-cognitive determinants such as those that predispose one to action (awareness factors and cues to action) and those that shift a person from being predisposed to action into an action state (clear action and coping planning and self-regulation skills) are not often investigated in the T1D systematic reviews. Overall, there is a gap in the systematic evidence that addresses the integrative socio-cognitive determinants of insulin adherence among late adolescents and young adults with T1D. Therefore, a comprehensive systematic evaluation of the evidence on the socio-cognitive determinants that predict adherence/non-adherence to insulin treatment among this age group is warranted. The findings will be important to guide patients’ consultations and diabetes self-management education (DSME) programs. They may also help to develop tailored insulin adherence improving interventions aimed at improving diabetes outcomes in patients with T1D. Therefore, this review aims to identify the key socio-cognitive determinants influencing adherence/non-adherence to insulin administration in late adolescents and young adults in the age range of 17–24 years with T1D. In order to ensure the systematic search of available literature, the Population, Exposure, Outcomes (PEO) strategy [64, 65] guided the formulation of the research question for this review.

## Methods

The methods of this systematic review have been developed and reported in compliance with the Preferred Reporting Items for Systematic Reviews and Meta-Analysis Protocols (PRISMA-P) [66] (see Annexure 1—PRISMA-P completed checklist) [67]. The study protocol is registered with the International Prospective Register of Systematic Reviews (PROSPERO) with ID: CRD42021233074.

## Inclusion and exclusion criteria

### Types of eligible studies

Studies will be selected for review if they were peer-reviewed cohort studies employing cross-sectional, longitudinal prospective, and retrospective cohort or mixed methods designs, published from 2000 to 2020 and written in English. Randomized and non-randomized comparative studies of interventions and studies investigating factors other than socio-cognitive determinants, such as only socio-demographic and/or only psychological factors will be excluded from the review. The reason for this is that  these studies do not address the research question of interest. Besides, previous reviews [5, 28, 68–70] have provided evidence for them. Commentaries, letters, and editorials will also be excluded.

### Population

Studies will be selected for review if they included adolescents and/or young adults in the age range of 17–24 years with clinical diagnosis of T1D. There will be no restrictions on the gender or ethnicity of participants. Since adherence is dynamic in nature [71], there will be no restrictions on the duration of diagnosis with T1D. Patients with a clinical diagnosis of comorbid conditions (e.g., depression, hypertension); people with cognitive impairments; drug or alcohol dependence; people who intentionally overdose for suicidal attempts, and pregnant women will be excluded because each of these population groups has conditions that affect the nature of insulin adherence behavior.

### Exposure variable(s)

Studies which investigated one or more of the socio-cognitive determinants associated with insulin adherence/non-adherence will be included. ICM guided the selection of the primary exposures of interest. Therefore, studies reporting on patient motivation, awareness of behavior and illness perception, awareness of risk perception, knowledge, cues of action, attitudes, self-efficacy, social influence, social norms, social modeling, action planning, coping planning, information, self-regulation skills, and service-related factors will be included in the review.

### Outcome variable(s)

Studies which used the participants’ adherence/non-adherence to insulin administration as the main outcome will be included. The secondary outcome will be the quantified association between any measured socio-cognitive determinant and adherence (if any). For more information about the outcome, please *see Appendix 3 in the extended data *[67]*.*

### Search strategy

A pre-specified search strategy will be used to search for studies in the electronic databases and citation indexes: PubMed, EMBASE, Web of Science, and PsycINFO databases. We aimed to achieve an optimal combination of databases to avoid missing relevant references [72]. The literature search will be limited to the English language and to articles published between 2000 to 2020 and will take place on 15 October 2021 until 1 January 2022. The emphasis was to complete study selection within approximately three months to ensure an-up to date systematic review before future studies on the same topic are conducted to avoid bias in the reported results [73]. A decision to identify studies only in the English language was undertaken due to time and budget resource limits [74, 75]. The decision on publication years was undertaken, considering that the scope of this review is relatively broad in terms of the socio-cognitive factors of interest and because too narrow or too broad inclusion criteria can lead to an ineffective screening process [70], therefore, we attempted to balance the thoroughness of searching published articles within a timeframe which is not too narrow (to minimize bias of missing articles) [75], nor too wide (to keep up-to-date with the most recent research evidence relating to systematic reviews in the social sciences [76] and the advancements in behavioral science in T1D) [77]. Reference lists from published studies and relevant reviews will be reviewed for additional papers not indexed in the databases searched, and when necessary, corresponding authors will be contacted for additional information [78].

A search strategy combining MeSH and EMTREE terms in PubMed and EMBASE, respectively, and free-text words (including term explosion) in the titles and abstracts will be used [79, 80]. The list of systematically formulated search strings containing four index terms: (1) population, (2) exposure, (3) outcomes, and (4) study design is peer reviewed by SJ using the Press peer review of electronic search strategies guidelines and any necessary adjustments will be made before running the search [81]. The PubMed search strategy is available as *Extended data* (Appendix 1) [67].

### Study selection

Study selection will follow the updated guideline for reporting systematic reviews (PRISMA 2020) [82] and will take place from 15 October  2021 to 1 January 2022. Duplicate records identified from database search will be first removed electronically in Endnote X9 following the method described by Bramer and colleagues (2016) [83]. Secondly, two researchers (HB and FS), working independently to minimize bias, will screen titles of all citations derived from the search. Thirdly, they will screen abstracts for eligibility. Finally, they will read and critically appraise the full text of each included study. During this process, the two researchers will discuss their findings; in case of uncertainty to either include or exclude the study, the full article will be read [84]. Furthermore, if any discrepancies in study selection between the two researchers still exist, a third researcher (LM) will be included in the discussion until consensus is reached.

### Assessment of methodological quality and risk of bias

Two separate reviewers will assess the quality of the included studies using The Joanna Briggs Institute (JBI) Critical Appraisal Checklist for Cohort Studies and JBI Critical Appraisal Checklist for Analytical Cross Sectional Studies [78]. The overall quality and risk of bias will be determined based on JBI guidelines [85]. A third reviewer will judge the disagreement, if any. These tools can be used to rigorously appraise the quality of observational studies by determining the extent to which a study has addressed the possibility of bias in its design, conduct and analysis [86]. JBI Critical Appraisal Checklists are depicted in Appendix 2 (*Extended data)* [67]. The number of positive answers to the questions will lead to the final score of the study. Studies will be classified as “high risk of bias (low quality)”, “moderate risk of bias (moderate quality)” and “low risk of bias (high quality)” if they score 0–3, 4–5, and 6–8 respectively, using the checklist for analytical cross-sectional studies, and 0–3, 4–7, and 8–11 respectively,  using the checklist for cohort studies. 

### Data extraction

We will use the population, exposure, outcomes, and study characteristics framework to extract data. Two reviewers will extract data independently (HB and LM ), a third independent reviewer (FS ) will resolve disagreements in data extraction until consensus. Data will be extracted using a standardized and piloted extraction form adapted from Cochrane Public Health Group Data Extraction and Assessment Template [87]. The following three types of data will be extracted from selected studies: a) study data, b) outcome data, and c) study quality. Study data will include:1) publication; 2) population; 3) study characteristics; 4) exposure; and 5) results and findings. Outcome data will relate to primary and secondary outcomes (see *Appendix 3 in the extended data*) [67]. Adherence is determined by using one or a combination of adherence to insulin measures (the commonly reported methods including the (adjusted) medication possession ratio, proportion of days covered (PDC), persistence, daily average consumption (DAC), and the Morisky Medication Adherence Scale (MMA) or by indirect methods such as using prescription claims, pharmacy/medical records or self-report questionnaires, visual analogue scale or by using cell-phone real-time assessment and computerized logbooks [17, 88, 89].

### Data synthesis

Meta-analysis will not be performed due to the expected heterogeneity across studies, because of the variety of socio-cognitive determinants used in eligibility criteria and/or methods used to measure insulin adherence. Hence, a qualitative narrative synthesis will be performed and summarized in a table of findings using GRADEpro, which will present the characteristics and quality of studies, and the outcomes of concern [90].

## Discussion

This systematic review will be performed to critically examine relevant literature and report the socio-cognitive determinants associated with adherence/non-adherence to insulin treatment in late adolescents and young adults with T1D. The findings will help design patient-centered interventions to promote adherence to insulin in this age group, and guide patients’ consultations and diabetes self-management education (DSME) programs.

Several systematic reviews have identified patient-perceived barriers as predictive of  non-adherence to self-care recommendations in patients with type 1 or type 2 diabetes [27, 62, 91]. However, unlike our proposed review, findings from previously published systematic reviews were not specific to late adolescents and young adults, nor to type 1 diabetes, and/or to insulin adherence. Given, the hazardous consequences of non-adherence to insulin on diabetes outcomes [18], in addition to the availability of evidence which shows that psychosocial factors such as beliefs, attitudes, and motivation have a greater influence on adherence than personality, metabolic, and demographic factors [92]. Moreover, patients' adherence to different domains of DSM is not uniform [6]. Therefore, our systematic review, grounded in theory, will fill this gap in the literature.

The proposed review is expected to have the following strengths. First, in order to enhance the performance and reporting of this systematic review, it will follow PRISMA 2020 guidelines [82], and will be conducted according to this reproducible protocol, which will provide evidence of the reliable conduct of the study [62, 91]. Second, four databases will be searched, which include a specialized database in the fields of behavioral sciences to avoid missing relevant references [72] and to minimize selection bias [75]. Third, the validated JBI checklist tools will be used to assess risk of bias of the included studies which address both the validity and reliability of a study [93]. However, the review is expected to have a few limitations. The various direct and indirect adherence measures to insulin treatment may hamper the comparison of adherence rates across studies. Other relevant evidence may be missed due to excluding Gray literature and articles published in a non-English language [75]. Despite these limitations, the proposed review will provide high level of systematic evidence on the subject of interest.

## Data Availability

Underlying data No data is associated with this article.
